# The Broader Economic Value of School Feeding Programs in Low- and Middle-Income Countries: Estimating the Multi-Sectoral Returns to Public Health, Human Capital, Social Protection, and the Local Economy

**DOI:** 10.3389/fpubh.2020.587046

**Published:** 2020-12-03

**Authors:** Stéphane Verguet, Paulina Limasalle, Averi Chakrabarti, Arif Husain, Carmen Burbano, Lesley Drake, Donald A. P. Bundy

**Affiliations:** ^1^Department of Global Health and Population, Harvard T.H. Chan School of Public Health, Boston, MA, United States; ^2^World Food Programme, Rome, Italy; ^3^Partnership for Child Development, Imperial College, London, United Kingdom; ^4^London School of Hygiene and Tropical Medicine, London, United Kingdom

**Keywords:** school feeding, benefit-cost analysis, economic evaluation, social protection, education

## Abstract

**Introduction:** Globally, there are 370 million children receiving school meals every day. Coverage is least in low-income countries, where the need is greatest and where program costs are viewed as high in comparison with the benefits to public health alone. Here we explore the policy implications of including the returns of school feeding to other sectors in an economic analysis.

**Methods:** We develop an economic evaluation methodology to estimate the costs and benefits of school feeding programs across four sectors: health and nutrition; education; social protection; and the local agricultural economy. We then apply this multi-sectoral benefit-cost analytical framework to school feeding programs in 14 countries (Botswana, Brazil, Cape Verde, Chile, Côte d'Ivoire, Ecuador, Ghana, India, Kenya, Mali, Mexico, Namibia, Nigeria, and South Africa) for which input data are readily available.

**Results:** Across the 14 countries, we estimate that 190 million schoolchildren benefit from school feeding programs, with total program budgets reaching USD11 billion per year. Estimated annual human capital returns are USD180 billion: USD24 billion from health and nutrition gains, and USD156 billion from education. In addition, school feeding programs offer annual social protection benefits of USD7 billion and gains to local agricultural economies worth USD23 billion.

**Conclusions:** This multi-sectoral analysis suggests that the overall benefits of school feeding are several times greater than the returns to public health alone, and that the overall benefit-cost ratio of school feeding programs could vary between 7 and 35, with particular sensitivity to the value of local wages. The scale of the findings suggests that school feeding programs are potentially much more cost-beneficial when viewed from the perspective of their multi-sectoral returns, and that it would be worthwhile following up with more detailed analyses at the national level to enhance the precision of these estimates.

## Introduction

Across the world, school feeding (SF) programs are implemented with the primary aims of addressing child hunger and nutritional deficits, and boosting school participation and learning. As of 2018, 117 countries report operating such schemes and as many as 370 million children receive school meals every day (1, 2). SF can take the form of hot meals or snacks prepared in schools or centralized community kitchens (3), or are incorporated into humanitarian assistance programs (4). The coverage of these programs can vary substantially: for instance, Ghana targets SF to government schools in deprived communities (5), whereas Brazil and India mandate the provision of meals in all public schools (6, 7). SF is also targeted to other vulnerable populations such as orphans, children with disabilities or former child soldiers (3). SF is often implemented as part of broader school health and nutrition programs, and is typically the most expensive component of these programs, requiring the daily provision of food throughout the school year (8).

Traditionally, the costs of SF have been compared with benefits in health and nutrition, or in education. However, SF programs have potential benefits spanning at least four major sectors: health, education, social protection, and agriculture. A recent review (9) suggests that a more realistic assessment of the returns to effective SF programs would include returns to outcomes in multiple domains. SF programs increase enrollment and reduce absenteeism which in turn enhance learning and support higher educational attainment. These effects are particularly strong for girls and young women since retaining girls in secondary education can increase educational achievement, reduce the risk of early marriage or inappropriate work, and limit exposure to major health risks, including HIV (9). School meal delivery platforms can also be used to provide other critical services such as deworming medication (3). Furthermore, SF can serve as a non-cash transfer equivalent to 10–15% of household income in low-income communities, and can thus serve as a strong incentive for parents to send children to school. In terms of economic effects, SF can generate sustainable and predictable demand for locally grown food and thereby positively impact the agricultural system and food supply, including the operations of small holder farmers. SF programs can incorporate bio-fortified foods, such as orange-flesh sweet potato and iron-fortified beans, in place of other vegetables, thereby boosting health benefits while simultaneously developing and maintaining local agricultural production (9). SF benefits are greater for the most vulnerable and marginalized, and so these programs are likely to be pro-poor and pro-woman (9).

Initially, motivations for social change, social protection, and poverty reduction were instrumental to the development and maintenance of national SF in a majority of countries (7, 10), and the programs were most often enacted and implemented by the education sector (11). More recently, the agricultural sector has taken a greater role in sustaining SF given the large potential of SF programs to support local food supply systems and agricultural production in developing countries, particularly in sub-Saharan Africa. With ~400 million schoolchildren receiving a school meal every day, inputs to SF programs represent a global market to the order of USD80 billion per annum (12).

Assessing the benefits and costs of SF programs in a comprehensive manner will demand accounting for all intersectoral benefits and costs. Hence, in this paper, building on recently published evidence (13, 14), we first develop an economic evaluation methodology to estimate the potential costs and benefits of SF programs across the health, education, social protection, and local agricultural sectors. Next, we apply a benefit-cost analysis framework and provide preliminary benefit-cost ratios for SF programs in select low- and middle-income countries (LMICs) spanning three world regions where input data are readily available. Our sample includes the country with the largest global SF program—India (7).

## Methods

We develop a benefit-cost analysis framework to conduct an economic evaluation of SF programs by tentatively accounting for effects across the fours sectors of health and nutrition, education, social protection, and the local agricultural economy.

We select countries that have data sources and key input parameters readily available to illustrate our methodology. Fourteen countries, whose SF programs have previously been studied in depth (15), were chosen: Botswana, Brazil, Cape Verde, Chile, Côte d'Ivoire, Ecuador, Ghana, India, Kenya, Mali, Mexico, Namibia, Nigeria, and South Africa^1^. The target population of the combined SF programs was the reported number of school students fed annually in each country (Table 1) (15).

**Table 1:** Estimated number of beneficiaries of school feeding programs in 14 countries, along with illustrative country-specific indicators (gross national income (GNI) per capita, under-five mortality rate).

**Country**	**Reported number of schoolchildren fed per year**	**GNI per capita (2018 USD)**	**Under-five mortality (per 1,000 live births)**
Botswana	333,000	7,985	37
Brazil	42,433,000	8,785	14
Cape Verde	85,000	3,550	20
Chile	1,850,000	15,270	7
Côte d'Ivoire	265,000	1,639	81
Ecuador	1,788,000	6,174	14
Ghana	1,739,000	2,159	48
India	113,600,000	1,990	37
Kenya	826,000	1,696	41
Mali	109,000	876	98
Mexico	6,100,000	9425	13
Namibia	300,000	5,810	40
Nigeria	9,301,000	1,935	120
South Africa	8,850,000	6,173	34

*Sources: Global School Feeding Sourcebook (2016) (15); World Bank (data pertain to the latest year for which data was available−2018) (17). Office of the Vice President of Nigeria (18)*.

Our benefit-cost analysis framework has five components: four components cover benefits (one component per sector) and one component captures the costs of SF programs. The benefit components include gains in health and nutrition, education, social protection, and local agricultural economies. The cost component encompasses the running costs of SF programs.

### Health and Nutrition Gains

The health and nutrition benefits of SF programs can be estimated by capturing potential reductions in anemia and worm infections. Our objective here is not to be precise or comprehensive. Rather, we choose two of the most prevalent health conditions that affect poor children in LMICs [e.g., anemia and soil-transmitted helminth (STH) infections] and that have been demonstrated to have long-run consequences for health and education (8, 19–25). We intend to convey the expected scale of effects that would emerge if SF programs were able to address just these two health conditions.

We first computed the number of cases of STH infections that would be averted by SF programs. We used the prevalence of any STH infection among 5–14 year-old (by world region (in 2015) for each country in our sample) (26) and the reported number of beneficiaries in schools (Table 1) to derive the likely number of beneficiaries with an STH infection (i.e., the avoidable STH burden) as in: Beneficiaries with STH = [Beneficiaries] ^*^ [STH prevalence]. Subsequently, we computed the impact of SF on reducing STH cases by utilizing the efficacy of low-cost, single-dose oral therapies in reducing STH infections when administered as part of SF's essential packages. SF effectiveness in reducing STH was assumed to be 90% (20). Using a simple static formulation, the number of STH cases averted could be estimated as [Beneficiaries with STH] ^*^ [SF effectiveness on STH]. Lastly, the STH cases averted were converted into disability-adjusted life years (DALYs) averted. For this, we used disability weights from the Global Burden of Disease (GDB) study (27), which were multiplied by the duration a child would have an STH infection (assuming a conservative duration of 5 years). We implemented a third of GBD's disability weight for intestinal nematode infections (symptomatic) as our best estimate given available data^2^. In other words, the estimated DALY burden per STH case was derived as: DALY_STH_ ~ 0.027 / 3 ^*^ 5 years ~ 0.045. Hence, DALYs averted by SF could be computed as: DALY_STH, av_ = [STH cases averted] ^*^ [DALY_STH_].

Similarly, for anemia-related benefits, we used the prevalence of anemia among 48–59 month-old across world regions (29) and the number of beneficiaries previously computed to derive: Beneficiaries with anemia = [Beneficiaries] ^*^ [Anemia prevalence]. For the effectiveness of SF in reducing anemia, we used randomized controlled trial (RCT) evidence from Uganda that showed that school meals and take-home rations would bring about a 17–20% point reduction in anemia prevalence in girls aged 10–13 years (30–32). Another RCT from India studied the impact of delivering iron-fortified salt through SF and found similar scales of effect (reduction of prevalence of any type of anemia by 9% points or 20% reduction) (33). It is noteworthy that high-quality studies from two different settings (i.e., from populations in different continents, sub-Saharan Africa and South Asia) show similar effect sizes for the population of interest (i.e., 5–14 year-old). We thus proceed assuming a 20% reduction as we intend to get a sense of the scale of the effect in this paper.^3^

The number of anemia cases averted was computed as: Anemia cases averted = [Beneficiaries with anemia] ^*^ [SF effectiveness on anemia]. We considered GBD disability weights of moderate cases of anemia (27), and also assumed a disease duration of 5 years (consistent with STH), in order to compute an average estimate. Thus, the estimated DALY burden per anemia case was derived as: DALY_A_ = 0.052 ^*^ 5 = 0.260. The anemia burden averted could then be estimated as: DALY_A, av_ = [Anemia cases averted] ^*^ [DALY_A_].

### Education Gains

Here, we considered the impact of SF on increasing school attendance (36). School meals could increase school attendance by 9% [drawing from a review of rigorously designed studies undertaken in LMICs over 1990–2015 (30, 37)], and this improvement can eventually increase future wages by 0.81% [one additional year of schooling leads to a 9% increase in future wages (38)].

We compute education gains per child as reflected by future wages (FW) earned in adult life; in doing so, we assumed an earning potential of 45 years of work (kicking in 5 years into the future, say from age 15 to age 60) discounted at 3% per year. Thus, we approximately derived: *FW* = W${}^{*}A^{*}\overset{49}{\underset{i=5}{\sum }}{\left(1+r\right)}^{i}$, where *W* is wage, *A* the impact of SF on wages (i.e., 0.81%), and *r* is the discount rate [3% per year, following economic evaluation standards (39)]. In the base case, we used countries' gross national income (GNI) per capita for the computation of FW gains. However, alternatives can be used (and were tested in sensitivity analyses; see Supplementary Appendix).

### Potential Social Protection Impact

We attempted to derive a crude money-metric value for the meals delivered to schoolchildren as an approximation of the social protection value conferred by SF programs. In particular, we sought to estimate the value of the transfer conferred to families with children participating in SF programs. About two-thirds (around 64%) of the SF budget is spent on food purchase (15). Therefore, we multiplied the annual SF cost per beneficiary by 0.64 and the number of beneficiaries per year to obtain the potential social protection impact of SF.

### Potential Impact to the Local Agricultural Economy

SF programs represent an opportunity to grow the local agricultural economy of LMICs. Hence, we quantified the potential benefits to the local agricultural sector (i.e., smallholder farmers) from implementing SF programs. To estimate the food needed per child per year, we used the daily ration for a home-grown school meal menu with a standardized kilocalorie (kcal) allowance of 700 per day [converted to kilograms (kg) using the composition of SF menus in selected LMICs including Kenya (15, 40)]. The 700 kcal allowance is consistent with Food and Agricultural Organization (FAO) normative standards (41, 42). Assuming 200 days of schooling over a year the amount of food to be produced is: [Beneficiaries] ^*^ [Food per beneficiary] ^*^ [200 days].

Subsequently, we sourced data on the total quantity of food that could be produced by a smallholder farmer in a year. We used data from farmers participating in the Purchase from Africans for Africa program (43)^4^ to compute the number of smallholder farmers to be mobilized to sustain local SF programs. Assuming a daily income per farmer per world region (44), we derived a monetary value for the local farming impact: [Farmers needed] ^*^ [Farmer income] ^*^ [200 days].

### Program Costs

We identified SF program costs per child per year [in 2012 USD for the 14 study countries (15)] to derive total running costs: SF cost = [Beneficiaries] ^*^ [SF cost per beneficiary]. No additional assumption was made regarding who would bear the cost of programs, whether it would be national authorities, regional governments, donor agencies, or non-governmental organizations. Furthermore, cost variations arising due to specific features of local settings could also not be considered due to lack of input data (15).

### Dashboard of Program Benefits and Costs

All four kinds of benefits of SF programs were either expressed or converted into money-metric values to enable comparison or aggregation. Education, social protection, and local farming impact are already expressed in money-metric terms (USD). Health and nutrition gains (expressed in terms of DALYs averted) had to be converted into USD value. For that purpose, consistent with the education gains, we assigned a value corresponding to the GNI per capita (per DALY averted).

We also conducted sensitivity analyses with: USD1000 or USD5000 (per DALY averted), following previous benefit-cost analyses undertaken for low-income and lower middle-income countries (45); minimum wages (43, 44, 46–49); and gross domestic product (GDP) per capita (see Supplementary Appendix). Finally, we computed aggregate benefit-cost ratios (BCR, i.e., the ratio of benefits to costs) that compared the total benefits to the total costs of SF programs when feasible. Results were reported at the region level and included ranges across countries within each region: Latin America (Brazil, Chile, Ecuador, Mexico); South Asia (India); and sub-Saharan Africa (Botswana, Cape Verde, Côte d'Ivoire, Ghana, Kenya, Mali, Namibia, Nigeria, and South Africa).

All computations were realized using Microsoft Excel 2016. All key input parameters used in the calculations are summarized in Tables 1, 2; and monetary terms are expressed in 2012 USD (as running costs of SF programs were measured in 2012 USD), unless otherwise stated.

**Table 2:** Key input parameters used in the comprehensive economic evaluation of school feeding programs.

**Input parameter**	**Value**	**Source**
Prevalence of STH infections (any type)	26.7, 26.4, and 25.5% in sub-Saharan Africa, South Asia, and Latin America, respectively.	(26)
Prevalence of anemia (any type)	63, 39, and 30% in West, Eastern, and Southern Africa; 49% in South Asia; and 25% in Latin America	Based on anemia prevalence among 48–59 month-old (29)
SF effectiveness on reducing STH prevalence	90%	Authors' assumption based on (20)
SF effectiveness on reducing anemia	20%	Authors' assumption based on (30, 32, 33)
SF effectiveness on increasing school attendance	9%	Authors' assumption based on (8)
DALY per STH case (years)	0.045	Authors' calculations based on (27)
DALY per anemia case (years)	0.260	Authors' calculations based on (27)
Increase in future wages gained via school feeding programs	0.81%	Authors' calculations based on (36–38)
School feeding program cost* (per child per year)	$10–$332	(15)
Food production per smallholder farmer per year	84 kg	(43)
Farmer wage (per day)**	$0.8 (sub-Saharan Africa); $2.7 (South Asia); $4.3 (Latin America)	(44)
Gross national income per capita*	$730–$14,350	(17)
Discount rate	3% per year	(39)

* *2012 USD*;

** *2009 International $*.

## Results

We first report the computed gains generated by SF programs on health and nutrition, and education, along with the program costs (Table 3). Next, we describe the potential impact in terms of social protection and local farming (Table 4).

**Table 3:** **(A)** Estimated number of soil-transmitted helminth (STH) cases averted, anemia (moderate) cases averted, and corresponding money-metric gains. **(B)** Estimated education gains in terms of future additional wages earned. **(C)** Costs.

**Outcome/region**	**Latin America**	**South Asia**	**Sub-Saharan Africa**	**All**
**(A) Health and nutrition**				
STH cases averted (millions)	12	27	5	44
Anemia cases averted (millions)	3	11	2	16
Money-metric value ($ millions)	14,431	6,082	3,049	23,561
Per capita money-metric value ($)	277 (126–335)	54 (N/A)	140 (32–200)	126 (32–335)
**(B) Education**				
Total future additional wages gained ($ millions)	109,161	29,668	17,332	156,161
Per capita future additional wages gained ($)	2,096 (951–2,532)	261 (N/A)	795 (129–1336)	833 (129–2,532)
**(C) Costs**				
Total costs ($ millions)	5,450	3,754	1,345	10,549
Per capita costs ($)	105 (41–332)	33 (N/A)	62 (10–104)	56 (10–332)

*School feeding programs across three world regions (captured via 14 countries). Note: money-metric gains are expressed in 2012 USD*.

**Table 4:** **(A)** Estimated potential social protection (income transfer) impact. **(B)** Estimated potential impact on local farming.

**Outcome/region**	**Latin America**	**South Asia**	**Sub-Saharan Africa**	**All**
**(A) Social protection**				
Income transfer of SF program ($)	3,488	2,403	861	6,752
SF food cost per child per year ($)	67 (26–212)	21 (N/A)	39 (6–67)	36 (6–212)
**(B) Farming impact**				
Total farming impact ($ millions)	14,190	8,512	784	23,486
Per capita farming impact ($)	273 (207–280)	75 (N/A)	36 (31–50)	125

*School feeding programs across three world regions (captured by 14 countries). Note: monetary terms are expressed in 2012 USD*.

For all outcomes examined, we found substantial heterogeneity based on variation in the underlying parameters. For example, the size of health and nutrition gains was contingent on the number of beneficiaries and the disease burdens alleviated. Meanwhile, the education gains, and the potential social protection and farming impacts depended on parameter values in local contexts such as the wage assumption, the annual cost of feeding a child, and the imputed income for smallholder farmers.

We summarize here the money-metric estimates identified. First, the total budgets of SF across the 14 countries examined would be about $10,549 million (Table 3). These costs would greatly vary across regions, with per capita costs of about $105 ($41–332 range) in Latin America, $33 in South Asia (India), and $62 ($10–104 range) in sub-Saharan Africa. Second, for health and nutrition, the gains would amount to roughly $23,561 million. Again, these benefits would greatly vary across settings, with per capita gains of $277 ($126–335) in Latin America, $54 in South Asia, and $140 ($32–200) in sub-Saharan Africa. Compared to SF costs ($10,549 million), this would yield a partial BCR of about 2.2, with large variations across settings: 2.6 (1.0–3.1) in Latin America, 1.6 in South Asia, and 2.3 (1.1–4.6) in sub-Saharan Africa. Third, SF programs would yield substantial education-related benefits (about $156,161 million), with varying per capita gains: $2,096 ($951–2,532) in Latin America, $261 in South Asia, and $795 ($129–1,336) in sub-Saharan Africa. The corresponding partial BCR for education gains would be of about 14.8, with the following variations: 20.0 (7.6–23.3) in Latin America, 7.9 in South Asia, and 12.8 (6.1–30.9) in sub-Saharan Africa. Fourth, with respect to social protection, the potential impact was estimated to be around $6,752 million (Table 4), which corresponds to the direct income transfer aspect of SF programs. The estimate would greatly vary across settings: per child transfers of $67 ($26–212) in Latin America, $21 in South Asia, and $39 ($6–67) in sub-Saharan Africa. Finally, for the local farming, the potential impact could amount to about $23,486 million—recall that this is roughly approximated as the income received by local smallholder farmers meeting the food demands of the SF programs. Again, the estimate would greatly vary across settings: per child impact of $273 ($207–280) in Latin America, $75 in South Asia, and $36 ($31–50) in sub-Saharan Africa.

In summary, across all 14 countries, total benefits across four sectors (health and nutrition; education; social protection; and local farming impact) could amount to as much as $210,710 million, while the total costs of all SF programs would be about $10,549 million. As a result, SF programs could yield returns on investment in the health and nutrition and education sectors of about 17 (yet with a wide range of 7–35 depending on the setting) while potentially providing social protection worth $6,752 and enabling growth of local economies worth $23,486 million. Importantly, our estimates are highly sensitive to the assumptions behind wage imputation: the BCR could decrease to as low as 3 to 1 with lower wages assigned (see Supplementary Tables 2, 3).

## Discussion

We presented in this paper a methodology to assess the broad benefits and costs of SF programs in 14 selected countries (13 LMICs and Chile). Our approach intends to capture, in money-metric terms, the substantial SF-driven impact from four sectors: health and nutrition, education, social protection, and local farming.

Our preliminary findings show that SF could yield substantial benefits for the program costs invested, with at least $7 of returns for every $1 invested in SF programs. This represents a large return on investment, comparable in magnitude to several of the best-buy intersectoral interventions identified through the Copenhagen Consensus exercise (45). Below, we explore the limitations of the methodology we used and discuss the main drivers of the identified BCRs, before considering implications for policy.

### Limitations of the Methodology

Our methodology presents a number of major limitations. First, there were important limitations in the data and estimates used. We focused on a sub-set of countries for which data were readily available; this was a convenience sample, and although we recognize that this was a deliberately diverse group we do not know whether it is representative. Thus, there are uncertainties around the extent to which the findings are externally valid. Furthermore, a number of key inputs involved assumptions, including the extent of local farming production, the money-metric valuation of school meals, and the relevance of the disability weights extracted from the GBD study. Second, we made a number of specific modeling choices in our estimation procedures. At the conceptual level, we relied on a static rather than dynamic model. In terms of more detailed choices: we made simplifying assumptions in the computation of future wages gained, specifically 45 years of future income and starting 5 years into the future; and we restricted our analysis to only four sectors, while there certainly are multiple other dimensions that could be considered on the benefit side (e.g., improvement of local political stability and conflict avoidance). Similarly, the impact on local farming production and the local economies did not account for pre-existing food production or the broader political economy landscape of food production. Likewise, we did not account for the amount of money local smallholder farmers could gain independently of SF programs purchasing their crop productions. Most importantly, we do not offer a summed total figure across the four sectors, and assume independent benefits across them. Rather, we speculate that an additive effect is a simpler (and perhaps probable) outcome to start with; and we argue that more research at the country level should be pursued (e.g., upper limits for the total benefits, possible cross correlations and independence across the sectors). This concept is indeed addressed in the FAO home-grown SF standards with reference to returns to education and agricultural economy (41) and in Alderman and Bundy (50) with reference to returns to human capital and social safety nets. To our knowledge, no other researchers have extended the argument to include all four sectors together [besides in (9)]. Also, we did not consider the geographical heterogeneity in health and education inputs, such as the distribution in disease burden or the distribution in educational attainment and quality of education received. The analysis assumes homogeneity and does not account for the distributional impact of SF programs across socioeconomic status, gender, and geographical settings within countries. As a result, our preliminary findings should be interpreted with caution, and further sensitivity analyses with additional countries should be conducted in the future.

### Identifying the Main Drivers and the Scale of Effect

Our analysis for all 14 countries suggests that the benefits are largely driven by the high rates of return on education. The estimated returns to health and nutrition, the impact on local economies by creating sustained and predictable demand for locally produced food, and the impact on social safety net gains from in-kind income transfers (i.e., the provision of school meals) are also important but much smaller than the returns to education.

Health and education during childhood and adolescence are key contributors to human capital. This is recognized in the analyses for the World Bank Human Capital Project (51), and can be conceptually measured by a single metric, especially Learning Adjusted Years of Schooling (LAYS) (52). SF appears to contribute directly to child human capital by improving health and more so indirectly by enhancing educational attainment. Overall, these human capital effects could lead to a BCR that varies from 7 to 35.

School meals also bring about significant positive returns along two other dimensions: social protection and the local agricultural economy. We measured these effects as returns to an income transfer and to food costs vs. food production costs, respectively. Each of these can be thought of as a ratio: in the former of income transfers as a proportion of SF costs, with the return being around 0.6; and in the latter of food costs vs. food production costs, of around 2.2 (0.3–5.1).

In earlier discussions of these data, we summed across the multiple returns and found that the overall return to SF programs could be in double figures if these several different benefits were taken together (9). While we still consider that to be reasonable conceptually (that is, that the multiple partial benefits for health, education, social protection, and the local agriculture economy can be combined additively to represent comprehensive benefits), we have concerns that simply summing the partial BCRs may not be a correct way to express the scale of the effects that are described by such different metrics. For now, therefore, we suggest that the more precise conclusion is that the return to human capital (i.e., from education and health improvements) represents a BCR varying between 7 and 35, and that SF programs also provide additional, substantial and independent returns to social protection, in the form of a transfer, and to the local agriculture economy, in the form of local purchasing equivalent to the value of the food provided. Future work should explore ways to express these very different types of returns in a combined metric.

The COVID-19 pandemic has brought new recognition of the role of SF programs in the health and development of schoolchildren. By March 2020, school closures across the world had resulted in some 1.5 billion children being excluded from education, and an estimated 390 million no longer receiving a meal at school. For many children this was the one guaranteed meal in the day, and the urgent efforts by governments and development agencies to replace the meal with cash transfers or take-home rations achieved at best partial success and at significantly higher cost. Even in rich countries the role of SF as a social safety net emerged strongly as a politically salient issue. With the growing back-to-school movement, SF and its role in incentivizing children to go to school, and parents to send them, has emerged as a near-universal element of the available international and national guidance frameworks (53).

### Conclusions and Next Steps

The intersectoral benefits of SF programs seem to promise an effective channel to promote socioeconomic development and to provide safety nets in LMICs. In this respect, further work should study additional dimensions in each of the four sectors examined in this report. For instance, in the health and nutrition sector, the effects of enhancing the nutrient contents of food provided to children and the intergenerational effects of SF programs warrant additional research. For the education sector, distributional issues regarding the location of schools and who benefits the most from SF programs (e.g., poor vs. rich), along with issues of female empowerment should be scrutinized to fully account for the potential equity benefits of SF programs. As for social protection, it will be important to think of how SF programs are integrated within the broader safety nets and poverty reduction policies specific to each country, and how SF programs may or may not promote opportunities for the poorest. Lastly, an important question will be how SF programs may encourage local food production and act as a catalyst for facilitating the growth of local economies in a sustainable manner.

The scale of the findings from this desk review suggest that SF programs are potentially much more cost-beneficial when viewed from the perspective of their multi-sectoral returns, and that it would be worthwhile following up with more detailed analyses at the national level. Furthermore, given the social determinants of health and the increasingly intersectoral nature of development policies in LMICs, and the recognition that schoolchildren should be placed at the center of the Sustainable Development Goals (54), it is essential that novel economic evaluation methods, such as the one used here, be developed to more fully reflect the multifaceted benefits and costs that these interventions imply across socioeconomic groups and in terms of non-health benefits (55, 56).

## Author Contributions

SV and DAPB conceptualized and initiated the study. LD, PL, and AC provided data. LD, CB, and AH provided advice. PL, AC, and SV did the research. PL and AC ran the analyses. SV wrote the first draft of the paper, which was reviewed by all authors.

## Conflict of Interest

CB and AH were employed by the United Nations World Food Programme while contributing to the study. The remaining authors declare that the research was conducted in the absence of any commercial or financial relationships that could be construed as a potential conflict of interest.

## Footnotes

^1^All countries in the sample were LMICs per World Bank income group classification (16) (except Chile) in 2012, the year for which country data were readily available.

^2^The burden of STH infection (number of worms per individual) varies non-linearly and is overdispersed. The burden is associated with morbidity, so morbidity distribution too is overdispersed. The assumption that disability affects one third of infections is an approximation for this. Life expectancy varies across the most prevalent 3 STH species (roundworm, whipworm and hookworm), and 5 years is also an approximation (28).

^3^Note however that RCTs from Burkina Faso and Laos (34, 35) where rations did not have multi-fortified foods did not demonstrate impact on anemia prevalence reduction (30).

^4^In the program (43), a total of 2,698 total tons of food was produced by 15,998 participating farmers over two years. Hence, 0.1686 = 169 kg were produced per farmer over 2 years or about 84 kg per farmer per year.
