# Supplementary material for: The Broader Economic Value of School Feeding Programs in Low- and Middle-Income Countries: Estimating the Multi-Sectoral Returns to Public Health, Human Capital, Social Protection, and the Local Economy
Source: Front Public Health. 2020 Dec 3;8:587046. doi: 10.3389/fpubh.2020.587046 (PMC7744483; doi:10.3389/fpubh.2020.587046)
Supplement: Supplementary file 1 [file Presentation_1.pdf]

Supplementary appendix

The broader economic value of school feeding programs in low- and middle-income countries: Estimating the multi-sectoral returns to public health, human capital, social protection and the local economy
by
Stéphane Verguet, Paulina Limasalle, Averi Chakrabarti, Arif Husain, Carmen Burbano, Lesley Drake, and Donald A.P. Bundy

We report in this supplementary appendix findings for our benefit-cost analysis when alternative assumptions for the imputed annual wages (in place of gross national income (GNI) per capita in the main text) were used, namely with: gross domestic product (GDP) per capita (2012 USD); a national income per capita set at $1,000 or $5,000 which approximates the World Bank cutoffs for low-income and lower-middle-income countries [1]; and minimum wages (Table S1). Notably, the values of $1,000 and $5,000 are consistent with previous cross-sectoral benefit-cost analyses conducted by the Copenhagen Consensus exercise [2].

Table S1. Gross domestic product (GDP) per capita and annual minimum wage for the 14 countries examined in our benefit-cost analysis.

Country	GDP per capita (2012 USD)	Minimum wage (2012 USD)
Botswana	7,070	959
Brazil	12,370	3,822
Cape Verde	3,448	1,218
Chile	15,352	4,625
Côte d'Ivoire	1,243	982
Ecuador	5,682	3,504
Ghana	1,588	741
India	1,444	936
Kenya	1,137	1,770
Mali	779	536
Mexico	10,242	1,195
Namibia	5,942	2,894
Nigeria	2,747	1,162
South Africa	7,501	4,002

To derive an estimate of minimum wage (MW) per country, we used a variety of estimates. For African countries, monthly average MW estimates from a study on wage schedules were used [3]. Botswana, Ghana, Kenya, and South Africa all had a reported monthly MW and these MWs were further multiplied by 12 to get annual estimates. The other African countries (Cape Verde, Côte d'Ivoire, Mali, Namibia, and Nigeria) did not have a reported MW, so the mean monthly MW for either low-income or lower-middle-income economies was used and multiplied by 12 to get an annual estimate. For Brazil, Chile, and Mexico, we used MW estimates as reported by the OECD [4]; for India, we used a proxy for the MW (137 Rupees per day) converted to USD drawing from [5]; and for Ecuador, we used a reported basic unified minimum wage in 2012 USD from [6].

Subsequently, the education benefits tied to future wages to be earned in adult life (assuming 45 years of income, kicking in 5 years into the future, discounted at 3% per year) could be computed with the following (consistent with the approach described in the main text):

$$FW = I_c * A * \sum_{i=5}^{49}(1+r)^i \ , \qquad \text{(S.1)}$$

where I_c is either country GDP per capita or minimum wage or \$1,000 or \$5,000. As a result, we could derive the increase in future wages capturing the educational benefits of SF programs (Table S2). Likewise, we could derive updated money-metric estimates for the health and nutrition gains (Table S3).

Table S2. Estimated educational benefits expressed in future wages gained through school feeding programs in three world regions (captured via 14 countries), with the use of different proxies for income per capita.

**Outcome/region**	**Latin America**	**South Asia**	**Sub-Saharan Africa**	**All**
GDP per capita (2012 USD)				
Total future additional wages gained ($ millions)	110,235	28,946	17,731	156,911
Per capita future additional wages gained ($)	2,117<br>(1,003 to 2,709)	255<br>(N/A)	813<br>(137 to 1,324)	837<br>(137 to 2,709)
$1,000 per capita				
Total future additional wages gained ($ millions)	9,189	20,046	3,848	33,083
Per capita future additional wages gained ($)	176	176	176	176
$5,000 per capita				
Total future additional wages gained ($ millions)	45,943	100,228	19,240	165,413
Per capita future additional wages gained ($)	880	880	880	880
Minimum wage (2012 USD)				
Total future additional wages gained ($ millions)	32,452	18,758	8,927	60,137
Per capita future additional wages gained ($)	623<br>(211 to 816)	165<br>(N/A)	409<br>(95 to 706)	321<br>(95 to 816)

Table S3. Estimated money-metric gains corresponding to the number of soil-transmitted helminth (STH) cases and anemia (moderate) cases averted. Three world regions (captured via 14 countries).

Outcome/region	Latin America	South Asia	Sub-Saharan Africa	All
GDP per capita (2012 USD)				
Total money-metric value ($ millions)	14,573	5,934	3,156	23,663
Per capita money-metric value ($)	280<br>(133 to 358)	52<br>(N/A)	145<br>(34 to 198)	126<br>(34 to 358)
$1,000 per capita				
Total money-metric value ($ millions)	1,215	4,109	777	6,101
Per capita money-metric value ($)	23	36	36<br>(26 to 44)	33<br>(23 to 44)
$5,000 per capita				
Total money-metric value ($ millions)	6,074	20,546	3,886	30,505
Per capita money-metric value ($)	117	181	178<br>(130 to 220)	165<br>(115 to 220)
Minimum wage (2012 USD)				
Total money-metric value ($ millions)	4,290	3,846	1,558	9,694
Per capita money-metric value ($)	82<br>(28 to 108)	34<br>(N/A)	71<br>(23 to 106)	52<br>(23 to 108)

Therefore, when using alternative values of income per capita, the total money-metric gains linked to education benefits could decrease to $33,083 million ($1,000 per wage) or $60,137 (minimum wage). This then leads to a partial benefit-cost ratio as low as 3.1 vis-à-vis the sole education sector. Likewise, the total money-metric gains linked to health and nutrition benefits could decrease to $6,101 million ($1,000 per wage) or $9,694 (minimum wage). This then leads to a partial benefit-cost ratio as low as 0.6 for the health and nutrition sector.

References

1. World Bank. World Bank Country and Lending Groups. Available at: https://datahelpdesk.worldbank.org/knowledgebase/articles/906519-world-bank-country-and-lending-groups
2. Copenhagen Consensus Center. Available at: https://www.copenhagenconsensus.com
3. Bhorat H, Kanbur R, Stanwix B. Minimum wages in sub-Saharan Africa: a primer. The World Bank Research Observer 2017; 32:21-74.
4. Organisation for Economic Co-Operation and Development. Real minimum wages. Available at: https://stats.oecd.org/Index.aspx?DataSetCode=RMW (accessed March 3, 2019).
5. Ministry of Labour and Employment (2017, June 7). India's revised national floor level minimum wage. Available at: https://labour.gov.in/gazette-notification
6. Wong SA. Minimum wage impacts on wages and hours worked of low-income workers in Ecuador. World Development 2019; 116:77-99.
